# Treatments for Ocular Diseases in Pregnancy and Breastfeeding: A Narrative Review

**DOI:** 10.3390/ph16101433

**Published:** 2023-10-09

**Authors:** Giuseppe Demarinis, Filippo Tatti, Andrea Taloni, Antonio Valentino Giugliano, Jesse Panthagani, James Myerscough, Enrico Peiretti, Giuseppe Giannaccare

**Affiliations:** 1 Department of Surgical Sciences, Eye Clinic, University of Cagliari, Via Ospedale 48, 09124 Cagliari, Italy; demarinis91@gmail.com (G.D.); filippotatti@gmail.com (F.T.); enripei@hotmail.com (E.P.); 2 Department of Ophthalmology, University ‘Magna Græcia’ of Catanzaro, Viale Europa, 88100 Catanzaro, Italy; andrea.taloni@studenti.unicz.it; 3 Department of Ophthalmology, Broomfield Hospital, Chelmsford CM1 7ET, UK; antonio.giugliano@nhs.net; 4 Department of Ophthalmology, Southend University Hospital, Southend-on-Sea SS0 0RY, UK; jesse.panthagani@nhs.net (J.P.); james.myerscough1@nhs.net (J.M.)

**Keywords:** pregnancy, eye, breastfeeding, eye drop, ocular diseases

## Abstract

Pregnancy is a medical condition in which the physiological changes in the maternal body and the potential impact on the developing fetus require a cautious approach in terms of drug administration. Individual treatment, a thorough assessment of the extent of the disease, and a broad knowledge of the therapeutic options and different routes of administration of ophthalmic drugs are essential to ensure the best possible results while minimizing risks. Although there are currently several routes of administration of drugs for the treatment of eye diseases, even with topical administration, there is a certain amount of systemic absorption that must be taken into account. Despite continuous developments and advances in ophthalmic drugs, no updated data are available on their safety profile in these contexts. The purpose of this review is both to summarize the current information on the safety of ophthalmic treatments during pregnancy and lactation and to provide a practical guide to the ophthalmologist for the treatment of eye diseases while minimizing harm to the developing fetus and addressing maternal health needs.

## 1. Introduction

Pregnancy represents a delicate balance, as it is necessary to consider the risks and benefits to the health of the mother and the developing fetus. Information on the safety and efficacy of therapies is usually not provided in randomized controlled trials, as these investigations are seldom feasible in pregnant women, owing to ethical concerns [1]. Therefore, the issue of the safety and efficacy of the use of drugs in pregnancy is ongoing and there is not enough information to reach an evidence-based answer [1]. Although the placenta acts as a “protective barrier” for the fetus, pharmacological and dietary substances may penetrate it, albeit in reduced concentrations. The teratogenic potential of most therapeutic agents in humans remains largely unknown. Animal studies have proven to be highly unreliable in demonstrating teratogenicity in humans, so the examples of false-positive and false-negative predictions based on these studies are well known [2]. In the few studies conducted in this field, it has been difficult to discern potential adverse effects on the fetus from normal complications attributable to pregnancy or underlying diseases [3].

Several routes of administration are available for the treatment of eye diseases: topical, periocular, intravitreal, and systemic [4]. Each of these modalities is chosen according to the type and severity of the pathology to be treated. Even when topical, intra-, or peribulbar administration is preferred, a certain amount of the drug may reach the systemic bloodstream [5].

In 1979, the US Food and Drug Administration (FDA) introduced the pregnancy letter risk categories “A, B, C, D, or X (potential fetal risk or positive evidence of fetal risk)” to help healthcare professionals assess the risks and benefits of using drugs during pregnancy and lactation [6]. However, several concerns have been raised because the risk categories are often misinterpreted [7]. In response to the need to update the risk categories, the FDA published a final rule entitled “Content and Format of Labeling for Human Prescription Drug and Biological Products: Requirements for Pregnancy and Lactation Labeling”, also known simply as the “Pregnancy and Lactation Labeling Rule” (PLLR), in December 2014 [8]. The new format of the PPLR includes information on pregnancy, breastfeeding, and exposure records, as well as a new subsection for women and men with reproductive potential. The latter gives information on pregnancy testing, contraception, and infertility in relation to drugs, when necessary [9]. To date, no topical ophthalmic drugs conform to the new labelling standard. Manufacturers of prescription drugs are required to relabel their drugs as the data on pregnant and lactating women become available.

This concise review provides updated information on the treatments of ocular diseases in pregnancy, including drug-specific risk evaluation and clinical utility based on published evidence.

## 2. Materials and Methods

We conducted a comprehensive literature review using the PubMed database. A thorough search of peer-reviewed articles was conducted in June 2023. Specifically, we developed a search strategy with different keywords reported in Table 1.

**Figure 1:**
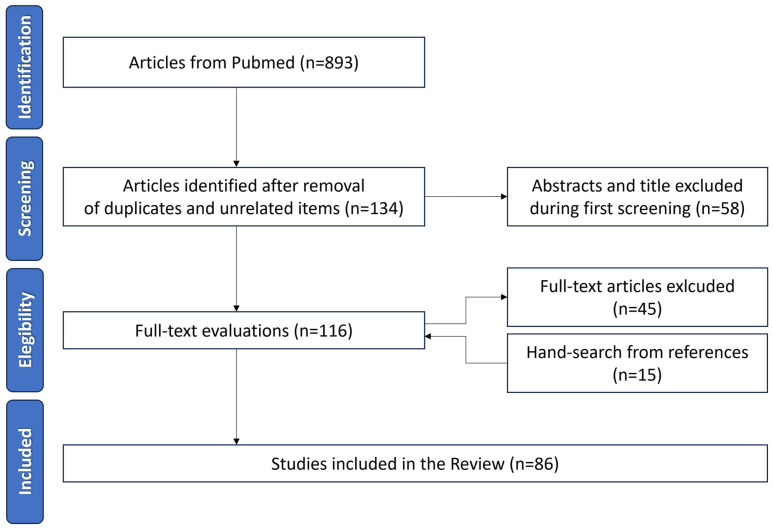
Preferred Reporting Items for a Systematic Review and Meta-Analyses (PRISMA) flowchart for the article selection process.

All identified studies were assessed by two authors (GP and FT) to determine the eligibility for inclusion in the analysis. The inclusion criteria were prospective and retrospective studies/series on humans and animals about eye medication and pregnancy, reviews on this topic, and articles in English, Spanish, and French. A total of 893 full-text articles were identified on PubMed, 669 of which were excluded after the first screening. The remaining 223 articles were evaluated for eligibility. After a full-text evaluation, 83 papers were used to evaluate the treatments in this review (Figure 1).

Although the review is based mainly on the articles published in the past 20 years (2000–2023), some articles published before 2000 were included because of the scarcity of research studies on this topic. Based on the available evidence, safety recommendations for the use of drugs during pregnancy and breastfeeding are provided in Table 2.

## 3. Ocular Drug Delivery

The eye can be roughly divided into two parts: anterior and posterior [47]. Depending on the part of the eye to be treated, several routes of drug administration are currently available. These are topical, systemic, intravitreal, and periocular [47]. Each modality is chosen according to the pathology to be treated while carefully weighing its advantages and disadvantages.

Topical administration, mainly eye drops, is an efficient treatment for diseases of the anterior segment of the eye [48]. It is a non-invasive route, but with low bioavailability (<5%) [48]. In fact, tear drainage, blinking, and naso-lacrimal drainage reduce the duration of drug bioavailability [49]. To overcome the insufficient corneal permeation and the short residence time in the ocular region, certain strategies can be implemented, such as frequent administration and/or high concentrations of the active agent, which may cause systemic side effects [49]. Indeed, there is ample evidence that between 34% and 79% of the administered drug is eliminated into the systemic circulation due to both the drainage of the naso-lacrimal duct and the high degree of conjunctival vascularization and its large surface area (16–18 cm^2^) [50,51].

Systemic administration is a coadjutant treatment or a second choice when an effective therapeutic concentration of the drug cannot be achieved via topical administration alone, such as for the posterior segment pathologies [47]. It consists of the administration of drugs via an oral, intravenous, or intramuscular route. Once the drug reaches the bloodstream, it can be absorbed into the ocular tissues via the conjunctival, choroidal, and episcleral vessels, although most drugs do not cross the main blood–ocular barrier [1]. 

Indeed, the tight junctions present in the blood–aqueous barrier and in the blood–retinal barrier lead to a drug bioavailability of less than 2% [47].

Intravitreal administration is the main treatment for diseases of the posterior segment of the eye. The drug is injected directly into the vitreous chamber, though it is a highly targeted drug route [51]. The major advantage is to achieve an effective concentration in the posterior segment of the eye, avoiding systemic exposure [51]. However, it is an invasive procedure and several major, albeit infrequent, drawbacks have been described: endophthalmitis, vitreous detachment, retinal hemorrhage, inflammation, high intraocular pressure, retinal toxicity, and the development of cataracts [51]. Moreover, after the injection, the drug could be eliminated via two different routes: the anterior route and the posterior route [51]. Concerning the former, the drug diffuses from the vitreous chamber to the posterior chamber and afterwards enters into the anterior chamber. At this point, the drugs are cleared via the turnover of the aqueous humor by the trabecular and uveoscleral outflow [51]. In the posterior route, the drugs permeate through the retina and are subsequently washed out of the choroidal bloodstream [51].

Periocular administration includes different administration routes based on the location and/or injection direction: subconjunctival, subTenon’s, retrobulbar, and peribulbar. A drug administered in this way can reach the posterior segment via three different pathways: the transscleral pathway; systemic circulation through the choroid; and the anterior pathway through the tear film, cornea, aqueous humor, and vitreous humor [5]. The advantages are a greater area of drug absorption and a high scleral permeability, while the disadvantages are poor drug bioavailability because of conjunctival blood and lymphatic circulation and the chance of an eye injury [51].

## 4. Anti-Infective Medications

Eye infections mainly involve the cornea, conjunctiva, and adnexal structures; in more severe cases, the infectious process can reach the internal structures such as the vitreous and retina. The location of the infected area describes the type of diagnosis [49]. Eye infections are generally treated topically (not systemically) by means of eye drops and injections, which provide very high and effective levels of anti-infectives in the target tissue [49]. Based on the causative agent, different drugs are currently available for obtaining an effective treatment.

### 4.1. Antibiotics

Topical antibiotics are the mainstay of the treatment of anterior segment bacterial infections, but limited evidence of their safety during pregnancy exists [10]. Indeed, few studies have been conducted about their use, so the therapeutic choice remains a risk-versus-benefit decision. Chloramphenicol is widely administered due to its broad spectrum of efficacy and relatively low cost [52]; however, its use in pregnancy is debated due to its documented serious side effects, including “gray baby syndrome” and “bone marrow suppression” [53]. Conversely, numerous clinical studies showed that chloramphenicol is safe in pregnancy if it is not in circulation at the time of delivery [54]. A recent study by Thomseth et al. investigated whether the exposure to topical chloramphenicol in the first trimester of pregnancy was associated with congenital malformations [11]. In this national cohort study, all women who received at least one prescription of chloramphenicol in the form of eye drops or ointment in Denmark were included [11]. A total of 966,372 births were examined from 1997 to 2011, and no association was found between the administration of chloramphenicol and major congenital malformations in the first trimester of pregnancy [11]. Conversely, during breastfeeding, the opinion of the experts suggests abstaining from its use, due to its reported side effects such as vomiting and meteorism [10]. 

For all remaining antibiotic eye drops or ointments, no studies on the ocular application have been conducted, and therefore no data on their toxicity in pregnancy and nursing are available. It is therefore suggested that the toxicity data reported after systemic administration should also be considered for topical ocular use.

Erythromycin, an antibiotic belonging to the macrolide group, is the oldest and the most used drug [12]. No reports have been found linking its use with congenital defects, but a hepatotoxicity has been described due to its placental transmission [10]. Moreover, it is considered by the American Academy of Pediatricians (AAP) and the World Health Organization (WHO) to be compatible with breast feeding [12,13]. For its safeness, erythromycin is one of the antibiotics of choice in the prophylaxis of ophthalmia neonatorum, a neonatal conjunctivitis caused by Chlamydia Trachomatis [55]. 

Polymyxin B, similar to Erythromycin, is considered one of the safest antibiotics. However, the placental transmission of the drug may cause side effects such as nephrotoxicity or neurotoxicity, although there are currently no reports describing drug-related congenital defects [10].

Aminoglycosides are another class of antibiotics commonly used in ophthalmology. Netilmicin, Neomycin, Gentamicin, and Tobramycin belong to this group. Currently, no studies have been conducted on the toxicity to the fetus after eye instillation during pregnancy, but it is widely recognized that these antibiotics are potentially nephrotoxic and ototoxic, particularly if administered in the first trimester of gestation [12].

Fluoroquinolones are the drugs of first choice in the treatment of corneal ulcers thanks to their properties and broad spectrum of action [56]. The use of oral fluoroquinolones in pregnancy has raised concerns due to their mechanism of action, which involves the alteration in DNA synthesis that could be a cause of organ agenesis, mutagenesis, and carcinogenesis in fetal tissues [14]. Yefet et al. conducted a meta-analysis evaluating the risk for fetal malformations and pregnancy complications following the exposure to quinolones during the first trimester of pregnancy. The authors concluded that quinolones are not associated with an increased risk of major malformations, stillbirths, preterm births, and low birth weight; however, as the information in the literature is limited, these antibiotics should not be used as a first-line therapy at this time [14]. Therefore, despite their undisputed efficacy in treating corneal ulcers, the use of fluoroquinolones in pregnancy is only recommended in the absence of alternatives.

Similarly, Tetracyclines should be prescribed with greater caution during pregnancy because of their well-known side effects: color alterations in deciduous teeth (with changes to brown) and the inhibition of bone growth [15]. These effects are mainly described in the case of administration during the second and third trimester of pregnancy, as well as in nursing women [12].

### 4.2. Antimycotics

The diagnostic and therapeutic pathway of fungal infection represents a challenge due to the difficulty of a prompt diagnosis and the limited availability of effective antifungal agents [57]. Although it is necessary to treat these infections, the maternal and fetal risk after a course of treatment is not well defined in humans and there is little or no data on this [16].

Antimycotics are divided into three different chemical categories: (i) polyenes; (ii) azoles, and (iii) 5-fluorocytosine (5FC) [58]. The route of drug administration depends on both the extent and the site of the infection [59]. The topical route of administration remains the more preferable one considering the high patient compliance and the ease of use despite the important drawback of low drug penetration, low residence time, the high frequency of administration, and ocular toxicity [57,59]. Unfortunately, it is not always possible to achieve an adequate concentration of antifungal treatment at the site of the injury [60]. In cases of a poor or unresponsive fungal eye infection to the topical treatment, the oral, parenteral, or intraocular route is chosen [59]. To date, Natamycin suspension is the only FDA-approved topical formulation for the treatment of ophthalmic fungal infections [57,61]. This drug is a fungicidal belonging to the class of polyenes [62]. Currently, no studies have been conducted on the ocular natamycin administration in humans during pregnancy; however, a gynecological retrospective study of the Hungarian malformation register, which examined 160 subjects treated with topical natamycin applied vaginally, revealed no evidence of an increased risk of malformations [17]. To date, according to the FDA, this drug should be used with caution if the potential benefits to the mother outweigh the potential risks to the unborn child [63].

Amphotericin B is the first polyene antifungal agent used for mycotic keratitis [62]. It has the largest number of published case reports documenting its use during pregnancy without adverse events to the fetus; thereby, it is considered the antifungal of choice in pregnancy for invasive fungal infections [16].

Azole is another category of antifungals widely used for the treatment and prevention of yeast and mold infections [16]. In this context, several case reports described the use of voriconazole for the treatment of fungal endophthalmitis [64,65]. Despite its good properties, voriconazole is not recommended in pregnancy due to the risk of fetal malformations in animal studies [16].

Finally, 5-fluorocytosine (5FC) concentrates in the eye and its use is highlighted for the treatment of candida endophthalmitis [66], but there are no adequate and well-controlled studies in pregnant females [16]. To be precise, in animal studies, 5FC was shown to be teratogenic in rats [16]. Consequently, it is contraindicated during pregnancy and breastfeeding [16].

### 4.3. Antivirals

To date, no studies have been conducted on the management of ocular herpetic infection during pregnancy. Samples et al., who first discussed the use of eye drops during pregnancy in 1988, stated that antivirals should not be prescribed to women during pregnancy as they are based on molecules that can intercalate in DNA and RNA [10]. However, their theory was based on the teratogenic effect found in some species of animals treated with the first antivirals of idoxuridine and vidarabine [10,67,68,69].

Subsequently, advances in topical ophthalmic antivirals have been made; acyclovir has been developed and its use in pregnancy has been widely investigated. In the cornerstone paper “Outcomes following systemic prenatal acyclovir exposure: Conclusions from the international acyclovir pregnancy registry, 1984–1999”, the authors investigated the use of oral acyclovir in 1246 pregnancies: 756 in the first trimester, 197 in the second trimester, and 291 in the third trimester [18]. Information gathered over a 15-year period indicates that the birth defects found following the antenatal exposure to acyclovir did not differ in the overall rate or type from those observed in the general population [18]. Pasternak et al. examined the use of acyclovir in 1561 women during the first trimester [19], confirming the previous findings of the lack of increased risk after assuming acyclovir [19]. 

Ganciclovir ophthalmic gel, one of the latest antivirals developed, is a further valuable tool for treating ocular herpes [70]. Again, there are no clinical trials that investigate the safety of this drug in pregnancy both in terms of oral and topical administration [71]. Consequently, the teratogenic risk during pregnancy cannot be excluded [20]. Similarly, the risk in nursing women is unknown; there is no current information on whether the drug can be absorbed by ocular tissues in sufficient quantity to be detectable in breast milk. Nevertheless, it is relevant to know that ganciclovir is secreted in the milk of laboratory animals; adverse effects have subsequently been observed in their offspring [20,21].

### 4.4. Antiamoebic

Acanthamoeba keratitis (AK) is a potentially blinding infection caused by protozoa found worldwide [72]. This disease is more commonly linked to poor hygienic practices when using contact lenses [72,73]. The topical application of biguanides and diamidines is the most popular anti-amoebic treatment for AK [73]. There is scarce evidence in the literature regarding their safety in pregnancy and breastfeeding. A recent case report described the occurrence of an AK in a 7-week pregnant patient [73]. Due to the catastrophic consequence of a corneal infection in the absence of a timely treatment and the unknown teratogenicity, the authors decided to implant lacrimal punctual plugs in the four lacrimal puncta and to commence the antiamoebic treatment [73]. This expedient made it possible, on the one hand, to use the drug during pregnancy and lactation and, on the other hand, to decrease the systemic concentrations of the drug, reducing the risk of harming the fetus [73]. 

## 5. Antihistamines

Anti-allergy drugs are used to treat inflammatory and allergic conjunctivitis. In general, information on the use of ophthalmic antihistamines in pregnancy is very limited: no epidemiological studies have been conducted on the effect of this class of drugs in human pregnancy [74]. The use of oral antihistamines during pregnancy has been very controversial due to the possible teratogenic effects of these drugs. None of the antihistamines available today have been categorized as safe during pregnancy [75].

## 6. Anti-Inflammatory Medications

Anti-inflammatory topical medications are therapeutically used for allergic or noninfectious conjunctivitis, dry eye, and acute anterior and posterior uveitis. Moreover, a co-administration of steroid drugs with antibiotics has also been frequently used in the therapy of bacterial ocular infections [76]. Although, on the one hand, it is possible to avoid or postpone the use of anti-inflammatory drops to reduce the symptoms of allergic, non-infectious, and infectious conjunctivitis, on the other hand, it is almost always necessary to treat anterior and posterior uveitis as they can irreparably impair vision [77]. Moreover, some anti-inflammatory medications were approved by the FDA for the treatment of diabetic macular edema (DME), macular edema following a branch or central retinal vein occlusion and posterior segment non-infectious uveitis [78]. In this context, previous studies demonstrated that uveitis improves during pregnancy, especially from the second trimester onwards, while the postpartum period is associated with a uveitis activity rebound [79]. 

### 6.1. Corticosteroids

The main pharmacological properties of corticosteroids are related to their anti-inflammatory effects; however, these medications can also cause the constriction of blood vessels, decreased cell proliferation, and immunosuppression. Several ocular corticosteroids are commercially available (betamethasone, fluorometholone, prednisolone, methylprednisolone, dexamethasone, hydrocortisone, and triamcinolone), and they may be locally administered under the following forms: topical, subconjunctival, periocular, and intravitreal [80]. During pregnancy, topical corticosteroids could be needed to treat inflammation of the conjunctiva, cornea, and anterior segment of the eye. Sub-conjunctival corticosteroids are frequently administered at the end of intraocular surgery; thus, excluding the emergency cases where ocular surgery cannot be postponed, this administration method is rare during pregnancy. Sub-tenon and peribulbar steroids are frequently preferred to treat ocular inflammatory conditions when systemic side effects are less desirable. The intravitreal route is typically used to stain the vitreous during vitreoretinal surgery, but more commonly for macular edema and uveitis [80]. To overcome the limitation of the rapid clearing of corticosteroids from the vitreous, slow-release preparations for intravitreal use are being developed. To date, there are three commercially available sustained-release intravitreal implants: Retisert (fluocinolone acetonide, Bausch & Lomb Incorporated, Bridgewater, NJ, USA), Iluvien (fluocinolone acetonide; Alimera Sciences, Alpharetta, GA, USA), and Ozurdex (dexamethasone; Allergan Inc., Irvine, CA, USA) [78].

Previous studies demonstrated that the systemic use of corticosteroids in animals during pregnancy induced cleft palate in fetuses [81]. Moreover, Ballard et al. found a direct correlation between the incidence of sex organ defects in mice and the dose of corticosteroids applied to the eyes [82]. 

In addition, the teratogenicity of the exposure to systemic corticosteroids in humans is controversial and there have been conflicting reports in the literature in recent years [81,83]. 

The fetotoxic effects of corticosteroids depend on the ability to cross the placenta [84]. The main enzyme that metabolizes corticosteroids is 11-beta-hydroxysteroid dehydrogenases-2. This enzyme converts hydrocortisone (the active form cortisol) into a biologically inactive cortisone, regulating the maternal cortisol that passes through the placenta. Considering the high metabolism in the placenta, hydrocortisone is presumed to be safe in pregnancy. However, a previous study demonstrated that the para of H-cortisol passed through the placenta without being metabolized [81].

The ability to cross the placental barrier varies among various corticosteroids: prednisolone crosses the placenta for 10% to 13%, whereas higher percentages were reported for betamethasone, methylprednisolone, and dexamethasone (30%, 45%, and 67%, respectively). Some drugs, such as fluticasone and budesonide, cross the placenta unhindered [81].

In a recent review concerning the use of topical dermatological corticosteroids, Ching-Chi Chi et al. showed evidence of an increased risk of low birth weight in pregnant women receiving more than 300 g of potent topical corticosteroids during pregnancy. However, the available studies did not support any causal relationship between the maternal use of topical corticosteroids and the other pregnancy outcomes, including the mode of delivery, congenital abnormality, preterm delivery, and fetal death [81]. 

No published studies have found a correlation between the administration of ophthalmic corticosteroids and teratogenicity in humans [26,85,86]. Despite the hematic absorption after the ocular administration, the lack of association is probably due to the low dosage of the drug used. In view of this, a previous article reported no significant difference between the serum levels of triamcinolone acetonide before and after an intravitreal high-dose injection of 20 to 25 mg [87]. Similarly, a pharmacokinetic study showed low plasma concentrations of dexamethasone at any time after a 0.7 mg intravitreal implantation [88]. Although there is no adequate literature evidence about the safety of intravitreally administered dexamethasone in pregnant women, Concillado et al. reported its use and safety profile during the various stages of pregnancy [22]. However, Ozurdex remained as not recommended during pregnancy and breast-feeding, unless the potential benefit justifies the potential risk.

With regard to topical administration, the general consensus is that prednisone should be preferred for the lower ability to cross the placenta. Moreover, previous studies demonstrated insignificant amounts of prednisone in breastmilk and no adverse effects have been reported in breastfed infants with maternal use of any corticosteroid during breastfeeding. Therefore, the corticosteroid therapy should be maintained or started in the post-partum period [23].

### 6.2. Nonsteroidal Anti-Inflammatory Drugs (NSAIDs)

In ophthalmic practice, topical NSAIDs therapy is not immunosuppressive and is commonly used for the treatment of postoperative inflammation and macular edema following cataract surgery [89]. Moreover, the use of topical NSAIDs has increased in the ophthalmic practice, mainly in the postoperative setting, because of their analgesic properties [90]. 

NSAIDs effects on the fetus and newborn depend upon the period of pregnancy in which the medication has been taken by the mother [91]. NSAID therapy during early pregnancy may be at a greater risk of having children with congenital anomalies [24]. On the other hand, the inhibition of prostaglandin synthesis caused by NSAID therapy after 28 gestation weeks may lead to the premature closure of the ductus arteriosus and renal impairment in the fetus [25,91]. Several studies do not consider topical medications as teratogenic, but it is preferable to avoid them in the third trimester of pregnancy. Moreover, due to the low concentration of breast milk, the AAP considers ibuprofen, naproxen, and indomethacin as compatible with breast-feeding [13].

### 6.3. Other Immunosuppressive and Anti-Inflammatory Drugs

As previously reported, exacerbations of inflammatory eye diseases could occur during pregnancy and in the post-partum period. Local injections of corticosteroids should be considered a valuable alternative in patients whose symptoms and inflammatory signs are not adequately controlled with topical therapy [23]. Several immunosuppressive drugs are contraindicated in pregnancy (methotrexate and alkylating agents), while others (azathioprine, and cyclosporin) may be considered for use in this period. However, most drugs are excluded from use in pregnancy not because of any proven teratogenicity, but because of the lack of available evidence of their safety for the fetus [23].

## 7. Mydriatics

These drops, which are typically used for diagnostic examination, are distinguished in two classes of drugs: parasympatholytic (atropine, cyclopentolate, and tropicamide) and sympathomimetic agents (phenylephrine). Mydriatic agents are applied topically as well as for the management of uveitis to achieve the dilatation of the pupil, paralyzing the iris sphincter muscles (tropicamide) or stimulating the iris dilator muscle (phenylephrine). Minor fetal malformations have been reported from the systemic use of phenylephrine, atropine, and homatropine, and there is a relative contraindication for their use during pregnancy [27,28]. Moreover, the sympathetic action of the phenylephrine could cause a general vasoconstriction, resulting in a renal failure of the newborn [92]. Generally, the use of these topical medications is considered safe; however, during the first three months of pregnancy, their application for an examination purpose is not recommended. In general, during pregnancy, tropicamide is preferred for its short duration of action, both as a diagnostic and as a therapeutic option [26].

## 8. Ocular Anti-Hypertensive Medications

Although glaucoma is an uncommon progressive disease of the optic nerve in women of childbearing age, its treatment and management may be challenging in pregnant or nursing women. Intraocular pressure (IOP) decreases in pregnancy, mostly in the second half, and this decrease may be even greater in pregnant women with pre-existing glaucoma or ocular hypertension [29,35,93]. IOP usually returns to its previous levels within 2–3 months after the delivery [94,95]. Given the IOP fluctuations, pregnant patients should be followed at least once each trimester and, in some cases, continuing the unchanged or increasing medications to control their IOP can be necessary.

### 8.1. Beta-Blockers

The most frequently used medications in the treatment of glaucoma are beta-blockers. These ophthalmic medications reduce eye pressure by decreasing the production of the aqueous humor. The systemic administration of beta-blockers during pregnancy could be associated with some adverse effects on the fetus and neonate, including premature labor pain, intrauterine growth retardation, bradycardia, polycythemia, apnea at birth, hypoglycemia, and hyperbilirubinemia [35,96]. However, according to the available data in the literature, there is no evidence to interrupt the topical beta-blocker use during pregnancy [26,35,97]. Indeed, it has been reported that the systemic burden of timolol administrated topically in both eyes is below the absorption of an oral dose of timolol [92]. Despite this point, with respect to the previous studies reporting issues in newborns exposed to beta-blockers near delivery, newborns exposed to these medications before birth should be observed after birth for bradycardia and other symptoms [35,98]. Otherwise, the medication could be suspended 2–3 days before delivery to avoid any adverse event in the infant [30].

A previous study demonstrated timolol presence in the breastmilk of a woman treated with topical medication, but this concentration was insignificant and unlikely to cause systemic side effects in the healthy breastfed infant [31].

### 8.2. Prostaglandin Analogs

Prostaglandin analogs reduce IOP by increasing the uveoscleral outflow. The use of this class of glaucoma medications in pregnancy is controversial. Several clinical trials demonstrated either no or rare systemic side effects attributed to topical prostaglandins [99,100,101]. Although prostaglandin analogs theoretically increase the uterine tone and may induce premature labor, the dosage used to stimulate abortion is much higher than the medication dosage in the ophthalmic formulation [35,98]. Despite some authors affirming that the use of this class of medications is contraindicated in pregnant women, it has been claimed that ocular prostaglandin analogs do not induce adverse effects in the fetus [32,35,102,103]. Nonetheless, given the theoretical risk of premature delivery, general caution is advised. Additionally, there is no evidence to support the use of these medications during lactation [33].

### 8.3. Alpha Agonist

Brimonidine is a selective α2-adrenergic agonist that increases the uveoscleral outflow and suppresses aqueous humor production. This medication is considered safe based on animal studies, although there are no well-controlled human studies ruling out any potential teratogenic effects. Moreover, central nervous system depression, somnolence, seizures, and apnea have been described as the side effects associated with brimonidine [34,104,105]. According to the blood−brain barrier penetration, and the possible presence in breast milk, the use of brimonidine poses a real risk of apnea or hypotension in infants. Thus, even if brimonidine is considered safe during pregnancy, it should be discontinued before labor and during breastfeeding to prevent potential fetal apnea in the infant [35].

### 8.4. Carbonic Anhydrase Inhibitors

The inhibition of carbonic anhydrase in the ciliary body decreases the production of the aqueous humor, and consequently, IOP. Systemic acetazolamide leads to frequent systemic side effects such as fatigue, paresthesia, depression, and metabolic disorders [90]. Oral carbonic anhydrase inhibitors, such as acetazolamide, are contraindicated late in pregnancy because they may cause electrolyte disorders, renal dysfunction, and pH derangement in the fetus [36,85]. In animal studies, a statistically lower fetal body weight was reported for orally applied high-dose brinzolamide, whereas no organ malformations were observed. Malformations of the vertebral bodies have instead been reported in rabbits exposed to dorzolamide during pregnancy [98]. However, no controlled reports of brinzolamide or dorzolamide exist in human pregnancy. In a study on five pregnant patients who received dorzolamide, no problems were observed in the neonates up to 2 years after birth [35].

There are no studies demonstrating the presence of these medications in human milk, so their safety in breastfeeding is unknown. Nevertheless, acetazolamide is approved by the AAP for use during lactation [13], due to the low plasma levels in infants exposed to the medication through breast milk [37].

## 9. Tear Substitutes

During pregnancy, hormonal, immunological, and vascular changes may affect the eye, modifying the physiology of the tear film, producing acinar cell destruction, and finally, resulting in dry eye disease (DED) [106,107]. Indeed, it has been shown that pregnant women have worse clinical signs of DED compared to age-matched non-pregnant women [108]. Moreover, previous studies have reported that the peak of ocular discomfort symptoms and DED signs occurred between the second and third trimester [109]. 

Pregnant women may be unsuitable for DED therapies such as topical immunomodulators and corticosteroids, whereas first-line tear substitutes are safe during pregnancy [26]. Specifically, a recent study demonstrated that the use of hyaluronate 0.1% alone, hyaluronate 0.3% alone, and diquafosol alone for DED in pregnant women was not associated with adverse neonatal outcomes [38].

## 10. Anesthetics

There is limited evidence about the effects of topical anesthetics during pregnancy or breastfeeding, while more comprehensive research is available on their systemic or local administration.

A retrospective multicenter study reported that from 11 to 23% of pregnant women have been exposed to local anesthetics during pregnancy, without any significant changes in the rate of fetal malformations [39]. However, the use of local anesthesia with bupivacaine and mepivacaine is not advisable, as it has been linked to the risk of prolonged bradycardia in the fetus [29]. In an animal study, the high doses of lidocaine administered with osmotic minipumps did not reveal any evidence of fetal harm [110]. Moreover, lidocaine concentrations in milk, during continuous intravenous infusion, or in high doses as a local anesthetic, are low, with poor absorption for the infant [40,41,111]. Hence, lidocaine is not expected to cause any adverse effects in fetuses or breastfed infants if locally administered. In summary, according to the FDA, most local anesthetics (lidocaine, prilocaine, and etidocaine) did not show teratogenic effects and may be safe to use, while bupivacaine and mepivacaine should be administered with caution during pregnancy, due to the potential risk of fetal bradycardia [39]. According to the available evidence, both lidocaine and bupivacaine are probably safe for breastfeeding when administered locally [41].

Conversely, although teratogenic effects have not been reported, no adequate data are available from the animal or human studies on the use of topical anesthetic eye drops, such as tetracaine, proparacaine, and oxybuprocaine, in pregnancy or breastfeeding. They should only be used during pregnancy if the potential benefits justify the potential risks to the fetus. Well-designed studies evaluating the teratogenic toxicity for anesthetic eye drops should be performed.

## 11. Anti-VEGF Injection

Anti-vascular endothelial growth factor (anti-VEGF) drugs are widely used for the treatment of several retinal disorders, with excellent results [112]. In young patients, their use could be justified for the treatment of choroidal neovascularization (CNV) from etiologies other than age-related macular degeneration, cystoid macular edema associated with uveitis, and diabetic retinopathy [113,114,115]. VEGF plays an important role in the regulation of vasculogenesis, neoangiogenesis, and vascular permeability [116]. In this context, it is widely described that VEGF is the main character in the maintenance of the fetal and placental vasculature. Reduced VEGF expression is teratogenic and linked to fetal loss in humans [42,117]. Consequently, spontaneous miscarriage in pregnant women may occur after the administration of anti-VEGF therapy [43]. Although its administration is via intravitreal injection, some of the drug may still reach the bloodstream through the trabecular and uveoscleral outflow and the choroidal blood stream, as described above [5]. Therefore, the ophthalmologist must take this into account when deciding whether this treatment should be recommended to a pregnant woman.

Evidence for the use of anti-VEGF in pregnancy is based only on case reports or small case series [42,43]. Three different anti-VEGFs, ranibizumab, bevacizumab, and aflibercept, are the most commonly used by physicians in daily practice [114]. Avery et al. showed that ranibizumab, bevacizumab, and aflibercept reach the bloodstream rapidly, but the former is eliminated very quickly, while the latter two take longer to be metabolized [44]. Furthermore, while ranibizumab had a minimal effect on plasma VEGF concentrations, bevacizumab and aflibercept significantly reduced VEGF in plasma [44].

Three different cases of miscarriage have been described after the intravitreal use of bevacizumab. Petrou et al. described two cases of miscarriage after the administration of bevacizumab during the first trimester of pregnancy [45]. Similarly, Gómez Ledesma et al. also reported a case of miscarriage after a single intravitreal injection [118]. Sullivan et al. described a case of pre-eclampsia. The injection was administered at 20 days of gestation and the other was 2 days before conception [119]. Conversely, other authors describe the administration of bevacizumab during pregnancy without adverse events [120,121,122].

Concerning ranibizumab, its use has been reported in a few case reports without any drawbacks [123,124], but one case report described a miscarriage after its administration during the first week of pregnancy [125].

To date, no published cases describe the human exposure to aflibercept during pregnancy, but in a recent pharmacovigilance study, its use was considered as potentially contraindicated during pregnancy [46].

In conclusion, there is limited clinical experience in the literature and, at present, it is not possible to establish a definite correlation between the use of anti-VEGFs and maternal–fetal complications. Therefore, their use should be carefully discussed with the patient while explaining the pros and cons. Furthermore, in patients with diabetic retinopathy, it is important to bear in mind the RESTORE study, which showed that visual acuity at 3 years in those who had delayed injections for 12 months was similar to that of those who had received early treatment. Therefore, stopping anti-VEGF treatment during pregnancy for 6–9 months may not seriously impair vision [126].

## 12. Conclusions

The safety of drug use in pregnancy is often an enigma. Few studies have been conducted to evaluate the effectiveness of these therapies in pregnant and breastfeeding women for both scientific and ethical reasons. Generally speaking, all medications should be avoided, if possible, in the first trimester because of the potentially higher risk of teratogenicity. From an ophthalmological point of view, the most used route of administration is the topical one; therefore, a small amount of the drug enters into circulation. For this reason, it is essential to always consider the risks described for systemically administered drugs. Each patient must be assessed individually, and the risk/benefit ratio of treatment must be carefully weighed. In addition, every pregnant woman receiving treatment must be fully informed of the possible side effects for herself and the fetus and must be closely monitored throughout treatment. Finally, in doubtful cases, or where a particular drug is strictly necessary, a multidisciplinary approach involving both the gynecologist and pharmacologist is strongly recommended to achieve both an effective treatment of the ocular problem and to safeguard the fetus.

## Figures and Tables

**Table 1:** Search strategy and keywords regarding “eye”, “pregnancy”, and “breast-feeding”.

Eye		Disease		Pregnancy
“eye” OR “ophthalmology” OR “eye drops” OR “ocular diseases” OR “ocular drugs” OR “ocular medication”	AND	“glaucoma” OR “uveitis” OR “ocular infection” OR “conjunctivitis” OR “keratitis” OR “retinopathy” OR “diabetic retinopathy” OR “maculopathy” OR “mydriatics” OR “anti-inflammatory” OR “ocular anesthesia”	AND	“pregnancy” OR “breastfeeding” OR lactation” OR “fetal risk”

**Table 2:** Summary of safety recommendations for drugs during pregnancy and breastfeeding. Drugs were considered contraindicated in pregnancy if no studies investigated their use on either animals or humans. If no study investigated the safety of drugs during lactation, the recommendation level for pregnancy was used when appropriate; otherwise, the drugs were marked as contraindicated.

Drug Family	Drug	Admin.	Ref.	Toxicity in Pregnancy	Recommendation	Ref.	Toxicity in Lactation	Recommendation
Anti-Infective Medications								
**Antibiotics**	Polymyxin B	Topical	[10]	Studied in systemic administration: no congenital defects. Possible nephrotoxicity.	Probably Safe	[10]	Studied in systemic administration: None	Safe
	Chloramphenicol	Topical	[11]	No congenital defects.	Safe	[10]	Vomiting, meteorism.	Contraindicated
	Erythromycin	Topical	[10]	Studied in systemic administration: no congenital defects. Possible hepatoxicity.	Probably Safe	[12,13]	Studied in systemic administration-	Safe
	Fluoroquinolones	Topical	[14]	Studied in systemic administration: no congenital defects. Mechanism of action based on alterations of DNA synthesis.	Caution	N/A	N/A	Caution
	Tetracyclines	Topical	[15]	Studied in systemic administration: color alteration of deciduous teeth; bone growth inhibition.	Caution	[12]	Studied in systemic administration: color alterations in deciduous teeth; bone growth inhibition.	Caution
**Antimycotics**	Amphotericin B	Topical	[16]	No congenital defects	Safe	[16]	No adverse effects.	Safe
	Voriconazole	Topical	[16]	Animal studies: Teratogenic.	Contraindicated	N/A	N/A	Contraindicated
	5-fluorocytosine	Systemic	[16]	Animal studies: Teratogenic.	Contraindicated	[16]	N/A	Contraindicated
	Natamycin	Topical	[17]	Studied in vaginal administration: No congenital defects.	Caution	N/A	N/A	Caution
**Antivirals**	Acyclovir	Topical	[18,19]	No congenital defects.	Safe	N/A	N/A	Probably Safe
	Ganciclovir	Topical	N/A	N/A	Contraindicated	[20,21]	Animal studies: adverse effects on the offspring.	Contraindicated
**Antimoebic**	Biguanides and diamidines	Systemic	N/A	N/A	Contraindicated	N/A	N/A	Contraindicated
**Antihistamines**								
**Antihistamines**		Systemic/ Topical	N/A	N/A	Contraindicated	N/A	N/A	Contraindicated
**Anti-inflammatory Medications**								
**Corticosteroids**	Dexamethasone	Intravitreal	[22]	No congenital defects. More evidence is required	Contraindicated	N/A	N/A	Contraindicated
	Prednisone	Topical	[23]	No congenital defects. 10–13% cross the placenta.	Caution	[23]	No adverse effects.	Probably Safe
**Immunosuppressive**	Methotrexate and alkylating agents	Systemic	[23]	Teratogenic.	Contraindicated	N/A	N/A	Contraindicated
	Azathioprine and cyclosporin	Systemic/Topical	N/A	N/A	Caution	N/A	N/A	Caution
**NSAIDs**	Ibuprofen, naproxen and indomethacin	Topical	[24,25]	Avoid early and in third trimester of pregnancy	Contraindicated	[13]	No adverse effects	Safe
**Mydriatics**								
**Parasympatholytic**	Tropicamide	Topical	[26]	Not recommended in 1st trimester	Probably Safe	N/A	N/A	Probably Safe
	Atropine and homatropine	Topical	[27,28]	Studied in systemic administration: Minor fetal malformations.	Caution	N/A	N/A	Caution
**Sympathomimetic**	Phenylephrine	Topical	[27]	Generally considered safe. Not recommended in 1st trimester. Case report of renal failure. Studied in systemic administration: Minor fetal malformations.	Caution	N/A	N/A	Caution
**Ocular Anti-Hypertensive Medications**								
**Beta-blockers**		Topical	[29,30]	Suspend 2–3 days before delivery or keep newborns under observation	Safe	[31]	No adverse effects	Safe
**Prostaglandin analogues**		Topical	[32]	Theoretically increase uterine tone and induce premature labor; unlikely with ophthalmic dosage	Caution	[33]	No evidence to support the use	Caution
**2-adrenergic agonists**		Topical	[34]	Animal studies: No congenital defects. To be discontinued before labor.	Safe	[35]	Risk of apnea or hypotension	Contraindicated
**Carbonic anhydrase inhibitors**		Topical	[36]	N/A	Contraindicated	[37]	N/A	Contraindicated
**Tear Substitutes**								
**Hyaluronate 0.1–0.3%**		Topical	[38]	No congenital defects	Safe	N/A	N/A	Safe
**Diquafosol**		Topical	[38]	No congenital defects	Safe	N/A	N/A	Safe
**Anesthetics**								
**Lidocaine, prilocaine and editocaine**		Local	[39]	No congenital defects	Probably Safe	[40]	No adverse effects.	Probably Safe
**Bupivacaine and mepivacaine**		Local	[29]	Fetal bradycardia.	Caution	[41]	No adverse effects for bupivacaine	Probably Safe
**Others**		Topical	N/A	N/A	Contraindicated	N/A	N/A	Contraindicated
**Anti-VEGF Injections**								
**Ranibizumab**		Intravitreal	[42]	Minimal effect on plasma VEGF, 1 case report of miscarriage during first week of pregnancy.	Caution	N/A	N/A	Probably Safe
**Bavacizumab**		Intravitreal	[43,44,45]	Several cases of miscarriage, 1 case of pre-eclampsia	Contraindicated	N/A	N/A	Contraindicated
**Aflibercept**		Intravitreal	[46]	N/A	Contraindicated	N/A	N/A	Contraindicated

Admin. = Administration, Ref. = References, NSAIDs = Nonsteroidal anti-inflammatory drugs.

## Data Availability

Data sharing not applicable. No new data were created or analyzed in this study.
